# Performance of a Short Version of the Everyday Cognition Scale (ECog-12) to Detect Cognitive Impairment

**DOI:** 10.14283/jpad.2024.109

**Published:** 2024-06-21

**Authors:** M. Manjavong, A. Diaz, M. T. Ashford, A. Aaronson, M. J. Miller, J. M. Kang, S. Mackin, R. Tank, B. Landavazo, D. Truran, S. T. Farias, M. Weiner, Rachel L. Nosheny, Alzheimer’s Disease Neuroimaging Initiative

**Affiliations:** 1 Division of Geriatric Medicine, Department of Internal Medicine, Faculty of Medicine, Khon Kaen University, Khon Kaen, 40002 Thailand; 2 Northern California Institute for Research and Education (NCIRE), 4150 Clement Street, 151NC, San Francisco, CA 94121-1563 USA; 3 Department of Veterans Affairs Medical Center, VA Advanced Imaging Research Center, 4150 Clement Street, San Francisco, California 94121 USA; 4 Department of Radiology and Biomedical Imaging, University of California San Francisco, 505 Parnassus Avenue, M-391, San Francisco, CA 94143-0628 USA; 5 Department of Psychiatry, Gil Medical Center, Gachon University College of Medicine, 21, Namdong-daero, 774 beon-gil, Namdong-gu, Incheon, 21565 Republic of Korea; 6 Department of Psychiatry, University of California, San Francisco, San Francisco, California 94143 USA; 7 Dementia Research Centre, UCL Institute of Neurology, University College London, London, WC1E 6BT UK; 8 Departments of Neurology, University of California Davis, 3160, Folsom Blvd, Sacramento, Davis, CA 95817 USA; 9 Department of Neurology, University of California San Francisco, 505 Parnassus Ave, San Francisco, CA 94143 USA; 10 Department of Medicine, University of California San Francisco, 533 Parnassus Ave, San Francisco, CA 94143 USA; 11 Department of Psychiatry, San Francisco VA Medical Center, University of California, San Francisco, 4150 Clement Street (114M), San Francisco, CA 94121 USA

**Keywords:** ECog-12, A short version ECog-12, Alzheimer’s disease, everyday cognition scale, dementia, MCI

## Abstract

**Background:**

The Everyday Cognition (ECog) 12-item scale, a functional decline measurement, can distinguish dementia from cognitively unimpaired (CU). Limited data compare ECog-12 performance by raters (self vs. informant) and scoring systems (average numeric vs. categorical grouping) to differentiate cognitive statuses.

**Objectives:**

To evaluate the performance of ECog-12 in differentiation cognitive statuses.

**Design:**

A cross-sectional diagnostic test study.

**Setting and Participants:**

Data from the Alzheimer’s Disease Neuroimaging Initiative (ADNI) study are analyzed. Participants were aged 55–90 years old divided into subgroups based on diagnostic criteria.

**Measurements:**

We evaluated ECog-12 performance across different diagnostic groups, such as CU vs cognitive impairment (CI; mild cognitive impairment (MCI), and dementia), and the association between ECog-12 and CI. This procedure was repeated for self- and partner (informant)-reports. Additionally, types of ECog scores were also assessed, where an average ECog score was calculated (continuous numeric) as well as a categorical grouping (“any occasional declined” or “any consistently declined”) based on item-level responses to ECog questions.

**Results:**

ECog-12 cut-off scores of 1.36 (self-reported) and 1.45 (partner-reported) distinguish CU from CI with AUC 0.7 and 0.78, respectively. Adding a memory-concern question improved self-reported-ECog AUC to 0.79. Self- and partner-reported “consistently-declined” ECog-12 categorical grouping provided AUC 0.69 and 0.78. The study partner reported ECog-12 showed a greater association with CI than self-reported, with odds ratios of 35.45 and 8.79, respectively.

**Conclusion:**

Study partner-reported ECog scores performed better than self-reported ECog-12 in differentiating cognitive statuses, and a higher study partner reported ECog score was a higher prognostic risk for CI. A memory concern question could enhance self-reported ECog-12 performance. This further emphasizes the need to obtain data from study partners for research and clinical practice.

**Electronic Supplementary Material:**

Supplementary material is available in the online version of this article at 10.14283/jpad.2024.109.

## Introduction

The recent development and FDA approval of disease-modifying treatments for Alzheimer’s disease (AD) increases the need for brief, efficient, and highly scalable tools that can be used to assess cognitive and functional status. Past studies (1–3) have shown the utility of Subjective Cognitive Decline (SCD) measures to identify older adults at risk, including instruments of subjective cognitive change and memory concerns.

The Everyday Cognition Scale (ECog) (4) is an instrument that measures the decline in everyday cognitive and functional abilities that map to six cognitive domains. It includes both self-report and informant/study partner-report versions. The original ECog, which consists of 39 items, has good test-retest reliability (r = 0.82) and external validity (4–7). It could significantly predict cognitive impairment progression (5, 6). ECog scoring outputs are usually examined as either (1) calculated average ECog scores with optimal cut-off and/or (2) group categorical assignment based on item-level response to ECog questions, such as “any consistent SCD” or “any occasional SCD” (1). The participants will be considered as “any occasional SCD” if any item in ECog-12 scores = 2, but none of the item scores ≥ 3. The participants would be considered to have “any consistent SCD” if any item on the ECog-12 was rated as 3 or higher (1). These scoring systems have shown utility in identifying those with cognitive impairment and those likely to undergo clinical progression (1).

In 2011, a shorter version of the ECog (ECog-12) was developed and validated (8) to reduce evaluation time while maintaining psychometric properties. Several studies have been conducted on the performance of the ECog-39 and ECog-12 scale (1, 5, 6). However, there has been no comparison between the continuous ECog-12 scale, which has an optimal cut-off point, and the categorical SCD defined by the ECog-12 scale. Additionally, there has been no comparison between the performance of ECog-12 from participants and their study partners to detect cognitive impairment at baseline. This is particularly important in participants with MCI or early AD who have positive amyloid pathology, as they represent a potential target population for an AD-modifying drug candidate. Since the ECog-12 consists of a simple, short questionnaire that is easy to administer in many settings (e.g., in clinic, remotely), we hypothesized that the ECog-12 scale might be useful as an efficient, first-step screening tool to identify older adults with cognitive impairment for AD research studies, clinical trials, and in clinical care settings.

The relationship between self- and study partnerreport ECog has also been found to be associated with important outcomes related to AD, although there is some inconsistency in results, with some showing agreement between self- and study partner-report and others showing discordance to be important (5, 9–14). When comparing self-reported ECog scores of the cognitively impaired participants with those reported by their study partners, several studies found lower scores for self-reports (11, 15). These lower scores from self-report were associated with poorer performance on memory tests and more AD biomarker evidence, such as CSF-based amyloid or tau biomarkers and brain volume (11, 12). Lower associations with disease outcomes for self-reported ECog scores (10) may be due to a person’s poor insight into their own cognition and function, called anosognosia. Therefore, because of poor insight, the ECog-12 rated by study partners may be a more accurate measure of impairment than the ECog-12 rated by self-report. Moreover, in most studies evaluating the association with cognitive performance, the original ECog score (ECog-39) was used (2, 16). Data compared the association with cognitive impairment between self and study partner reported that the ECog-12 was limited.

In addition to subjective reports of functional decline, self-reporting a memory concern increases the likelihood of progression from SCD to MCI (1). Therefore, abnormalities from the ECog scale added to the self-report memory concern may increase the performance and prognostic risk to detect cognitively impaired patients.

The primary aim of this study was to test the hypothesis that the performance of the ECog-12 can accurately distinguish five different diagnostic groups, including 1) cognitively unimpaired (CU) from cognitively impaired (CI; those with MCI and AD) older adults, 2) CU vs MCI, 3) CU vs. AD, 4) AD vs. MCI, and 5) CI with confirmed brain amyloid pathology vs others (CU&CI without amyloid pathology). Diagnoses were provided by study physicians, serving as the gold standard. For the fifth group (detecting CI with confirmed brain amyloid pathology), we aimed to investigate the ability of ECog to identify those who would be suitable candidates to receive currently approved AD disease modifying therapeutics. This includes those with evidence for CI and elevated brain amyloid, but not those who are CU (regardless of amyloid status). In this study, an amyloid-PET scan or CSF biomarkers was used to detect brain amyloid pathology.

The secondary objectives were to compare performance between self and study-partner ECog-12, to evaluate multiple ECog scoring outputs (e.g., average score, categorized score), to examine whether different measurement parameters can enhance sensitivity to group discrimination, to examine the predictive power of self-reported memory concern when added to ECog-12, to compare the performance of ECog-12 and ECog-39 in detecting cognitive impairment, and to evaluate the association between ECog-12 and cognitive impairment.

## Methods

This was a cross-sectional study to evaluate the performance of the self-and study partner-report short version of ECog (ECog-12) in detecting cognitive impairment at baseline. For the gold standard of cognitive impairment detection, this study used a final diagnosis at baseline from ADNI data, which was a clinical diagnosis from a medically qualified professional.

### Subjects and study setting

Data used in this study were obtained from the Alzheimer’s Disease Neuroimaging Initiative (ADNI) database (adni.loni.usc.edu). The ADNI was launched in 2004 as a public-private partnership led by Principal Investigator Michael W. Weiner, MD. The primary goal of ADNI has been to validate biomarkers for clinical trials, specifically whether serial magnetic resonance imaging (MRI), positron emission tomography (PET), biofluid-based biomarkers (genetics, CSF, plasma), and clinical and neuropsychological assessment can be combined to measure the progression of mild cognitive impairment (MCI) and mild dementia.

The participants (n = 1593) included in this study were all ADNI participants who had available self- and study partner report ECog scores. They were aged between 55–90 years and clinically diagnosed as CU (n = 666), MCI (n = 707), or mild dementia (n = 220) at baseline visit. Participants who had major psychiatric and neurological diseases were excluded from the study. The complete inclusion and exclusion criteria can be downloaded from https://adni.loni.usc.edu/methods/documents/.

### Procedure

During the baseline visit, participants were required to complete the ECog-39 questionnaire. Their study partners were asked to respond to the same questionnaire in reference to the participant’s cognitive and functional status. Additionally, participants were asked if they were concerned about their memory or thinking abilities.

The clinicians evaluated the participants and diagnosed them as CU, MCI, or mild dementia during the same visit. The baseline characteristics were recorded, including age, gender, years of education, marital status, race, and APOE4 status.

#### ECog-12

The ECog-12 score in this study was derived from the ECog-39 data. The ECog-39 consists of 39 functional assessment questions correlated to specific neuropsychological domains: memory, language, visuospatial function, planning, organization, and divided attention. Participants and their study partners completed the ECog-39 separately. They were asked to rate changes in level of participants’ current functioning in the last 10 years. Then they could answer each question by providing scores ranging from 1–4, with 1 indicating no change in ability over 10 years, 2 indicating occasionally perform the task worse, 3 indicating consistently perform little worse on task than 10 years ago, and 4 indicating participants perform the task much worse than 10 years ago. Moreover, the respondents can select the “don’t know” option if they are uncertain about their answer. Based on the prior study, the ECog-12 data was derived by choosing 2 items per domain from ECog-39 (8). The questions of ECog-12 are shown in Appendix 1.

In this study, the ECog-12 was scored by both continuous and categorized methods. For the continuous score, we calculated the average ECog-12 score by the sum of the total score divided by the number of items answered (this accounts for items with missing responses or the rater indicating the “don’t know”). The minimum and maximum score were 1 and 4. For the categorical assignment, participants were grouped into 3 categories based on ECog item-level responses. The participants would be defined as “any occasional SCD” in the case any item of ECog-12 was rated = 2, but none of the items was rated ≥ 3. The participants would be defined as having “any consistent SCD” in the case any item of ECog-12 was rated at least 3 (1). Participants whose itemlevel responses were all “1” were grouped as “stable/not declining”.

#### Memory concern question

Only participants were asked to answer whether they were concerned about their memory or not. The question was, “Are you concerned that you have a memory or other thinking problem?” and they can reply as “Yes” or “No”. Out of a total of 1593, 1378 participants responded to this question.

### Diagnosis criteria

#### Cognitively unimpaired and cognitively impaired participants

In this study, participants were classified as CU, MCI, or dementia based on clinical judgment from study physicians at the baseline visit. The final diagnoses were given by the study physicians based on overall cognitive and functional assessment. The term “cognitively impaired participants” refers to MCI or mild dementia participants.

#### Cognitive impairment with positive amyloid pathology

We identified a group of participants who would be suitable candidates to receive new disease-modifying therapy for AD (17). The participants were diagnosed with MCI or mild dementia and had brain amyloid pathology evidence. The amyloid pathology evidence was evaluated by using an amyloid-PET scan or CSF biomarkers in cases where a PET scan was not available. During the baseline visit, an amyloid-PET scan and lumbar puncture for CSF analysis were performed. For the amyloid-PET scan, the standardized uptake value ratio (SUVR) was converted to the Centiloid scale (CL) to standardize differences caused by radiotracer types. Amyloid-PET scans where the CL was at least 18.5, indicates the presence of amyloid pathology (18). For participants who did not have PET scan results, the CSF biomarkers were used. CSF Aβ42 < 980 pg/mL and ptau181/Aβ42 ratio ≥ 0.025, indicating the presence of amyloid pathology (19–21).

### Statistical analyses

The overall cohort was divided into subgroups based on diagnostic criteria (CN, MCI, or mild dementia), and descriptive statistics were tabulated for baseline ECog-12, participant age, education, gender, marital status, race, and APOE4 status. Baseline differences between groups were evaluated using independent Kruskal-Wallis tests for continuous variables and Pearson’s chi-squared tests for categorical factors, with a significance threshold a = 0.05.

Logistic regression was used to evaluate the performance of the ECog-12 score with and without a memory concern question to classify diagnostic groups. The models included differentiating between CU vs. Cognitive Impairment (CI; MCI and mild dementia), CU vs MCI, mild dementia vs. MCI, CI with confirmed brain amyloid pathology vs others (CU and CI without amyloid pathology), and CU vs. mild dementia. The cutoff points used to dichotomize ECOG-12 were calculated using the cutpointr package (22), using kernel smoothing methods to optimize Youden’s J index. AUCs and other summary statistics are reported based on ordinary logistic regression models.

To evaluate the association between ECog-12 and cognitive impairment, we first used a logistic regression model with ECog-12 score as the sole independent variable and fit for the dichotomous response variable. We also used a multivariable logistic regression model with other participant characteristics included as covariates. This procedure was repeated twice for the self-report and study partner-report versions of the ECog 12.

## Results

### Participants

A total of 1593 participants and 1571 study partners were included in this study. The median age of participants was 71.4 years old (IQR 66.7, 76.8). Among participants, 666 (41.8%) were CU, 707 (44.4%) were diagnosed with MCI, and 220 (13.8%) were diagnosed with mild dementia. The average ECog-12 score of participants ranged from 1 to 4, and the median score was 1.46 (IQR 1.18, 1.83), while the ECog-12 score of study partners raged from 1 to 3.92 and the median was 1.33 (IQR 1.08, 2). For the ECog-39 score, it ranged from 1 to 3.86 and from 1 to 3.95 for participants and study partners, respectively. The median score of ECog-39 in participants was 1.5 (IQR 1.24, 1.9), and in study partners was 1.35 (IQR 1.1, 2). Out of 1593 participants, 69% or 951 individuals responded that they had memory concerns. The participants’ baseline characteristics are shown in Table 1, separated by cognitive status: CU, MCI, and mild dementia.

**Table 1:** Baseline characteristics of participants

**Characteristic**	**CU (n = 666)**	**MCI (n = 707)**	**dementia (n= 220)**	**p-value**
Age; mean (sd)	70.99 (6.7)	71.65 (7.5)	74.25 (8.29)	<0.001
Male; n (%)	268 (40%)	390 (55%)	132 (60%)	<0.001
Education years; mean (sd)	16.68 (2.36)	16.17 (2.58)	15.7 (2.61)	<0.001
Race; n (%)				<0.001
- American Indian/Alaskan	2 (0.3%)	2 (0.3%)	0	
- Asian	26 (3.9%)	10 (1.4%)	8 (3.6%)	
- Black	82 (12%)	42 (5.9%)	14 (6.4%)	
- White	538 (81%)	632 (89%)	196 (89%)	
- More than one	14 (2.1%)	11 (1.6%)	2 (0.9%)	
Unknown	4 (0.6%)	8 (1.1%)	0	
Marital status; n (%)				<0.001
- Married	458 (69%)	531 (75%)	192 (87%)	
- Others	208 (31%)	176 (25%)	28 (23%)	
APOE4 allele; n (%)				<0.001
- Negative	387 (68%)	326 (53%)	64 (32%)	
- 1 allele	166 (29%)	228 (37%)	93 (47%)	
- 2 allele	17 (3%)	63 (10%)	42 (21%)	
CDR-SB; median (IQR)	0 (0,0)	1 (0.5,2)	4.5 (3.5, 5.5)	< 0.001
Positive brain amyloid evidence by PET or CSF; n (%)	192 (30%)	354 (52%)	178 (84%)	<0.001
Memory concern; n (%)	269 (43%)	495 (91%)	187 (88%)	<0.001
Average ECog-12 of participants; mean (sd)	1.35 (0.34)	1.77 (0.58)	1.91 (0.63)	<0.001
Average ECog-12 of study partners; mean (sd)	1.18 (0.27) (n = 653)	1.73 (0.62) (n = 697)	2.68 (0.69) (n = 221)	<0.001
”Any consistent SCD” categorization of ECog-12 for participant response; n (%)	224 (34%)	498 (70%)	167 (76%)	<0.001
”Any consistent SCD” categorization of ECog-12 for study partner response; n (%)	98 (15%)	435(62.4%)	206 (94.5%)	<0.001
”Any occasional SCD” and “any consistent SCD” groups combined ECog-12 for participant response; n (%)	573 (86%)	658 (97%)	213 (97%)	<0.001
”Any occasional SCD” and “any consistent SCD” groups combined ECog-12 for study partner response; n (%)	390 (59.8%)	643 (92.3%)	217 (99.54%)	<0.001
”Any Consistent SCD” group ECog-12 for participants with positive memory concern complaint; n (%)	148 (24%)	373 (69%)	151 (71%)	<0.001

CU, cognitively unimpaired participants; MCI, Mild Cognitive Impairment; AD, Alzheimer’s disease; SCD, Subjective Cognitive Decline; CDR-SB, Clinical Dementia Rating Scale sum of box score; IQR, interquartile range. P-values represent differences between diagnostic groups based on the Kruskal-Wallis rank sum test for continuous variables or the Chi-square test for categorical variables. P-values < 0.05 indicate significant differences.

### Performance of ECog-12

#### To differentiate cognitive impairment and cognitively unimpaired group

The performance of each cut-off point of the average ECog-12 score from both participants and study partners is shown in Appendix 2A. The cut-off point at ≥ 1.36 of ECog-12 from participants provided the best Youden index (0.41) and AUC (0.7) to distinguish CU from cognitively impaired. For the average score from the study partner, ECog-12 ≥ 1.45 showed the best Youden index (0.57) and AUC (0.78).

Table 2 and Appendix 2B demonstrated the performance of the categorized grouping ECog-12 and the average ECog-12 scores with optimal cut-off points from participants and study partners, and the ECog-12 scores from participants combined with memory concern in detecting cognitive impairment. The average score of ECog-12 with the optimal cut-off point of the study partner showed higher performance in detecting cognitive impairment than the average self-report score (AUC 0.78 and 0.7, respectively). For self-report ECog-12, adding the memory concern question increased the AUC to 0.79 (95%CI 0.77–0.82). For the categorized ECog-12 score, the participants’ score showed a fair performance in the detection of cognitive impairment, but the performance increased to moderate after adding on the memory concern question (AUC 0.73, 95%CI 0.7–0.75). Although “any consistent SCD” ECog-12 of study partners showed moderate performance in differentiation (AUC 0.78, 95%CI 0.76–0.8), it provided higher performance than those from participants.

**Table 2:** Performance of ECog-12 to differentiate cognitive statuses

**ECog-12 status**	**CI vs CU AUC (95%CI)**	**MCI vs CU AUC (95%CI)**	**dementia vs MCI AUC (95%CI)**	**CI with amyloid pathology vs others (CU&CI without amyloid pathology) AUC (95%CI)**
Average ECog-12				
ECog-12 of pt*	CP ≥ 1.36 AUC = 0.7 (0.68-0.73)	CP ≥ 1.37 AUC = 0.7 (0.67-0.72)	CP ≥ 1.7 AUC = 0.57 (0.53-0.61)	CP ≥ 1.36 AUC = 0.65 (0.63-0.68)
Ecog-12 of sp*	CP ≥ 1.45 AUC = 0.78 (0.76–0.8)	CP ≥ 1.27 AUC = 0.75 (0.72–0.77)	CP ≥ 1.9 AUC = 0.77 (0.74–0.8)	CP ≥ 1.45 AUC = 0.75 (0.73–0.78)
ECog-12 of pt with memory concern **	AUC = 0.79 (0.77–0.82)	AUC = 0.8 (0.77–0.82)	AUC = 0.49 (0.46–0.53)	AUC = 0.71 (0.69–0.73)
Categorized ECog-12				
Any consistent SCD categorization of ECog-12 for pt response	AUC = 0.69 (0.67–0.71)	AUC = 0.68 (0.67–0.71)	AUC = 0.53 (0.49-0.56)	AUC = 0.65 (0.62–0.67)
Any consistent SCD” group ECog-12 for pt with positive memory concern complaint vs others***	AUC = 0.73 (0.7–0.75)	AUC = 0.72 (0.67–0.75)	AUC = 0.51 (0.48-0.55)	AUC = 0.68 (0.66–0.71)
Any consistent SCD Ecog-12 of pt + concern vs none of both	AUC = 0.79 (0.77–0.82)	AUC = 0.8 (0.77–0.82)	AUC =0.49 (0.46-0.51)	AUC = 0.71 (0.69–0.74)
Any consistent SCD categorization of ECog-12 for sp response	AUC = 0.78 (0.76–0.8)	AUC = 0.74 (0.71–0.76)	AUC = 0.66 (0.64-0.68)	AUC = 0.74 (0.72–0.76)

CP, cutpoint; AUC, Area Under the Curve of the “Receiver Operating Characteristic” curve; pt, self-participant; sp, study partner; CI’ cognitive impaired participants; CU, cognitively unimpaired participants; * Dichotomous score on each cutpoint; ** Performance of ECog-12 combined with positive concern question compared with none of high ECog and memory concern; ***AUC derived from the performance of participants who had any consistent SCD ECog-12 score and positive memory concern complaint compared to those without any consistent SCD in ECog12 and memory concern complaint, as well as those with positive only one of each.

#### To differentiate MCI and cognitively unimpaired group

The performance of the ECog-12 scores from both participants and study partners in detecting MCI from normal cognition is shown in Table 2 and Appendix 3A and 3B. The optimal cut-off point for ECog-12 from participants was ≥ 1.37 (Youden index 0.39 and AUC 0.7), while the optimal cut-off point of study partners’ ECog-12 was ≥ 1.27 (Youden index 0.49 and AUC 0.75), the result was shown in Appendix 3A. The average ECog-12 of self-participants at the cut-point ≥ 1.37 combined with memory concern had the best AUC (0.8, 95%CI 0.77–0.82). Excluding the memory concern question, the performance of an average ECog-12 from study partners showed better performance compared with participants’ ECog-12 in detection (AUC = 0.75 and 0.7, respectively). Similarly, the performance of “any consistent SCD” ECog-12 from study partners was higher than the participants’ (AUC = 0.74 and 0.68). After adding the memory concern to any consistent SCD ECog-12 of participants, the AUC was increased to 0.72 and 0.8, as shown in Table 2.

#### To differentiate mild dementia and MCI

The performance of the ECog-12 scores from both participants and study partners in detecting mild dementia from MCI is shown in Table 2 and Appendix 4A and 4B. All average and categorized ECog-12 scores from self-participants had poor performance in differentiation (AUC ranged from 0.49 to 0.57). However, the performance of average and any consistent SCD ECog-12 from study partners showed moderate to good (AUC 0.77) and fair (AUC 0.66) performance in detection.

#### To differentiate cognitive impairment with confirmed amyloid pathology participants (AD drug candidate participants) and others (non-AD drug candidate participants)

The performance of the ECog-12 scores from both participants and study partners in detecting cognitive impairment with confirmed amyloid pathology is shown in Table 2 and Appendix 5A and 5B. Similar to other results reported here, ECog-12 of study partners with both average score (AUC = 0.75) and any consistent SCD ECog-12 category (AUC = 0.74) showed better performance than self-report ECog (AUC = 0.65) in detecting drug candidate patients from others.

#### To differentiate mild dementia and cognitively unimpaired group

The performance of the ECog-12 scores from both participants and study partners in detecting mild dementia from normal cognition is shown in Appendix 6A and 6B. Both average and any consistent SCD ECog-12 scores from study partners had excellent performance in differentiating mild dementia from cognitively unimpaired (AUC 0.93 and 0.9, respectively), while the average and any consistent SCD ECog-12 scores of participants with and without memory concern showed only moderate performance (AUC ranged from 0.71–0.78).

#### Comparison of ECog scoring outputs

For all models, the ECog-12 and ECog-39 performed similarly in distinguishing diagnostic groups; the results of ECog-39 performance are shown in Appendix 7. Continuous scores from both self-report and study-partner report ECog demonstrated a similar performance as the categorized ECog scale in distinguishing most analytic groups, results as shown in Table 2. However, to distinguish between mild dementia and MCI participants, the continuous ECog-12 score of study partners performed better than the categorical score (AUC 0.77 and 0.66, respectively).

The association between ECog-12 and cognitive impairment is shown in Table 3. Higher self-report ECog-12 was associated with higher odds of cognitive impairment in univariable (crude odds ratio 10, 95%CI 7.4–13.76) and adjusted models (adjusted odds ratio 8.79, 95%CI 6.32–12.43). Higher study partner-report ECog-12 was associated with higher odds of cognitive impairment, with an adjusted odds ratio of 35.45 (95%CI 22.41–58.03)

**Table 3:** Association between ECog-12 and cognitive impairment (MCI and AD)

**Variables**	**Univariate analysis**		Multivariable analysis (model 1*)		Multivariable analysis (model 2**)	
	**Crude OR (95%CI)**	**P value**	**Adjusted OR (95%CI)**	**P value**	**Adjusted OR (95%CI)**	**P value**
Average ECog-12 of participants	10 (7.4–13.76)	<0.001	8.79 (6.32–12.43)	<0.001		
Average ECog-12 of study partner	38.01 (25.12–59.18)	<0.001			35.45 (22.41–58.03)	< 0.001
Age	1.02 (1.01–1.04)	<0.001	1.01 (0.99–1.03)	0.44	0.99 (0.97–1.01)	0.22
Male gender	1.91 (1.56–2.34)	<0.001	2.05 (1.58–2.67)	<0.001	1.71 (1.28–2.28)	0.003
Education	0.95 (0.87–0.94)	<0.001	0.88 (0.83–0.92)	<0.001	0.94 (0.88–0.99)	0.02
Race (white)	1.99 (1.45–2.65)	<0.001	1.5 (0.97–2.34)	0.07	1.18 (0.74–1.91)	0.49
Married	1.62 (1.29–2.03)	<0.001	1.37(1.02–1.84)	0.04	0.82 (0.59–1.13)	0.23
APOE4 positive	2.16 (1.8–2.6)	<0.001	2.07 (1.69–2.56)	<0.001	1.69 (1.34–2.13)	<0.001
AUC			0.795		0.87	

*Model1: association of average ECog-12 score from self-participants and cognitive impairment adjusted with age, gender, educational years, race, marital status, and APOE4 status; **Model2: association of average ECog-12 score from study partners and cognitive impairment adjusted with age, gender, educational years, race, marital status, and APOE4 status

## Discussion

The major findings of this study were: First, the ECog-12 rated by study partners had higher performance than the ECog-12 rated by participants themselves to detect cognitive impairment. Second, adding a memory concern question improved the ability of self-report ECog to distinguish diagnostic groups. Third, average scores from both self-report and study-partner reports demonstrated a similar performance as the categorized scale in most analytic groups. However, to distinguish between mild dementia and MCI participants, the average ECog-12 score of study partners showed good performance, while the categorized ECog-12 from study partners and the ECog-12 scores from the participant’s data had low classification performance. Fourth, the average and “consistently declined” ECog-12 scores from study partners provide fair sensitivity to detect cognitively impaired participants who had positive amyloid pathology (AD modifying drug candidates), and the average ECog-12 from participants added to the memory concern question provided an excellent sensitivity with moderate AUC in detection. Fifth, the ECog-12 showed a comparable performance to the ECog-39.

Our study found that the study partner-report ECog 12 was better than the self-report ECog 12 score for distinguishing all diagnostic groups examined. AUC for study partner-report ECog ranged from 0.75–0.93, whereas AUC for self-report ECog ranges from 0.57 to 0.74. The difference between self- and study partnerreport ECog was greatest in the CU vs mild dementia models (AUC 0.93 for study partner ECog, AUC 0.74 for self-report ECog). In addition, after adjusting for participants’ demographics, the ECog-12 from study partners showed a stronger association with cognitive impairment than self-report ECog [adjusted OR for study partner 35.45 (22.41–58.03) and for self-participant 8.79 (95%CI 6.32–12.43)], as shown in Table 3. These results are consistent with several prior cross-sectional studies that revealed informant-reported SCD was better than self-reported SCD in distinguishing different diagnostic groups (2). A study from the original ECog (ECog-39) showed that ECog-39, rated by study partners, discriminates cognitive statuses better than self-reports.(10) The higher accuracy of the study partners’ ECog-12 than the self-participants’ could be explained by the cognitively impaired participants tending to underestimate their cognitive and memory function, called anosognosia, which is the impairment of insight or denial into their illnesses (23–25). Compared to other existing scales from a prior systematic review and meta-analysis study (26), the Mini-Mental State Examination (MMSE) provided a combined sensitivity of 0.81 (95%CI 0.78–0.84) and specificity of 0.89 (95%CI 0.87–0.91) for detecting dementia, while study partner-reported ECog-12 at a cut of point ≥ 1.67 from our study showed higher sensitivity (0.93) and specificity (0.95). For detecting MCI, the Montreal Cognitive Assessment (MoCA) outperformed ECog-12 with a sensitivity of 0.89 and specificity of 0.75 (26). However, the ECog-12 demonstrated superior advantages in the remote evaluation compared to the MoCA. A previous study conducted by ADNI and the Brain Health Registry demonstrated that Online Everyday Cognition is highly consistent with in-clinic Everyday Cognition (27).

Moreover, our results support the anosognosia hypothesis of the cognitively impaired participants. In the dementia group, the study partners rated higher average ECog-12 scores than participants. In contrast, the mean ECog-12 of the study partners was lower than the participants in CU and MCI groups. These results were similar to previous studies using ECog-39, which also found lower study partner scores in those groups (15, 28, 29). A previous study, from the Brain Health Registry data, evaluated the accuracy of online self- and study partner-reported ECog-39 to distinguish MCI from CU participants. The result of that study revealed that the mean ECog-39 score by online study partner report was higher than the self-report ECog-39 in the Aβ positive MCI group (mean ECog: study partner = 2.03 (SD 0.61) and self-report = 1.88 (0.67)). While the mean ECog-39 by study partner report was lower than the self-report in normal control and Aβ negative MCI (15).

Comparing the categorized and continuous average ECog-12 scale, our results demonstrated that the average score from both self- and study partner reports provided a similar performance as the categorized scale in all analytic groups except between dementia and MCI participants (AUC from any consistent SCD group and average ECog-12 of study partners were 0.66 and 0.77, respectively). These results were quite different from the study by Dr. van Harten et al. (2018), which evaluated the association between the risk of MCI progression and abnormal ECog scores. They reported that the average ECog score with optimal cut-point showed slightly lower strength of association with the risk of MCI progression compared to any consistent SCD (any response ≥ 3 compared to all responses < 3 of the ECog scale), hazard ratios 2.06 and 2.45, respectively (1). However, the methodology of prior and current studies was different. The current study was a cross-sectional study to detect cognitive impairment at baseline, but the prior was a longitudinal study to predict cognitive impairment progression.

The self-report memory concern question was also important. Our study showed that adding a memory concern question to ECog-12 from participants increased the accuracy of detecting cognitive impairment in most diagnostic groups. The results supported that participants of this study still have an awareness of their symptoms. These were similar to the prior population-based cohort study results that showed a subjective memory complaint associated with a faster cognitive decline (30) and could discriminate MCI and early AD from cognitively unimpaired participants (31). In addition, several longitudinal studies suggested that the subjective memory concern may be an early manifestation of AD and MCI (32–35). However, adding the memory concern question decreased accuracy in distinguishing mild dementia and MCI participants in the current study. That is because the presentation of memory concern is common in both mild dementia and MCI patients, particularly the amnestic MCI, which is the most common subtype (36, 37). Thus, adding a memory concern question couldn’t improve the performance of ECog-12 in distinguishing between dementia and MCI. In this study, only participants with mild dementia were included. If individuals with more severe stages of dementia had also been included, the results may have differed.

A novel aspect of this study was evaluating the ECog for identifying older adults who would be suitable candidates for AD therapeutics targeting amyloid, which have been approved for treatment for those with MCI and mild AD dementia. Our study showed that average and “consistently declined” ECog-12 scores from study partners provide fair sensitivity to detect participants with cognitive impairment who were amyloid positive (sensitivity 0.77 and 0.78, respectively). However, the average ECog-12 from participants added to the memory concern question provided an excellent sensitivity with moderate AUC to detect the drug-candidate participants (sensitivity 95% and AUC 0.71 see Appendix 5A and 5B). Therefore, the self-report ECog-12 plus memory concern question might be an appropriate and easy initial screening tool to identify individuals who may be candidates for AD disease-modifying medication.

Our study showed similar performance between average ECog-12 and ECog-39 from both self-participants and study partners among all diagnostic groups, as shown in Table 2 and Appendix 7. Thus, ECog-12 has comparable psychometric properties to the original version but with fewer items and a shorter evaluation time. These results were similar to a previous study, which demonstrated that ECog-12 had an excellent and comparable performance to ECog-39 in differentiating cognitive impairment from cognitively unimpaired (AUC; ECog-39 = 0.91, ECog-12 = 0.91) and between dementia from cognitively unimpaired (AUC; ECog-39 = 0.96, ECog-12 = 0.95) (8). Compared to the prior study in 2011, the cut points of Ecog-12 form study partners in our study to detect cognitive impairment are lower likely because participants in the current study have better cognitive performance than those in the previous one (median CDR-SB scores in MCI and mild dementia in the current study were 1 and 4.5, and in the prior study were 1.87 and 6.71) (8).

This is the first study to compare the performance of different scoring methods (average score vs. categorical grouping) of a short ECog version (Ecog-12) and to compare performance between self- and study partner-ECog-12 for detecting cognitive impairment at baseline (particularly AD medication candidate participants).

These findings support that ECog-12 can be used in multiple settings to facilitate dementia research, trials, and clinical care. It can be used to help screen participants for clinical trials and observational studies. It can also be used by clinicians to identify patients with possible cognitive impairment, who should undergo further screening. Additionally, the ECog-12 scale could be useful as an efficient, first-step screening tool to identify participants who are suitable candidates for AD disease-modifying therapy. We encourage using study partner-reported ECog-12, both the average score at the cutoff score ≥ of 1.45 and a categorical grouping (any consistently declined) to efficiently screen and assess older adults for research studies, clinical trials, and clinical care. The self-reported ECog-12 can be used as well, but self-reported ECog-12 alone provided AUCs less than 0.8. Therefore, the memory concern question should be evaluated by adding to self-reported ECog-12 to increase diagnostic accuracy.

This study has several limitations. The ADNI study design may limit the generalizability of results to other populations and settings. ADNI is an observational study using a sample of convenience that lacks ethnocultural and educational diversity. We did not use separate “training” and “validation” cohorts or sub-samples to externally validate the cut-offs we established. Thus, the predictive performance of our models may be overestimate. Further studies are being planned to validate the results in other cohorts. The dementia participants included in this study had mild dementia, so the result could not be generalized to moderate and severe stages of dementia. The Ecog-12 in this study derived from the original version of ECog (ECog-39), so some items might not be updated and relevant to the current real-world activity (such as the capability of technology usage). The current ADNI4 study will evaluate the performance of ECog-12 derived from the new version of ECog (ECog-II) (38) in detecting cognitive impairment and may show better results (39). Other dementia subtypes and other AD stages of participants should be enrolled in future studies to generalize and expand the utility of this tool to other populations.

## Electronic Supplementary Material

Supplementary material, approximately 44.2 KB.

## Notes

*Ethical standards:* The ADNI protocols have been approved by all Institutional Review Boards of the participating institutions. The data used for these analyses were collected only from volunteers who provided written informed consent.
