# Supplementary material for: Performance of a Short Version of the Everyday Cognition Scale (ECog-12) to Detect Cognitive Impairment
Source: J Prev Alzheimers Dis. 2024 Jun 21;11(6):1741–50. doi: 10.14283/jpad.2024.109 (PMC11573828; doi:10.14283/jpad.2024.109)
Supplement: Supplementary file 1 — Supplementary material, approximately 44.2 KB. [file 42414_2024_109_MOESM1_ESM.docx]

**supplementary materials**

**Appendix1**

ECog-12 questions

| Questions | Domain |
| --- | --- |
| Remembering where he/she have placed objects. | Memory |
| Remembering the current date or day of the week. | Memory |
| Communicating thoughts in a conversation | Language |
| Understanding spoken directions or instructions. | Language |
| Reading a map and helping with directions when someone else is driving. | Visuospatial |
| Finding his/her way around a house visited many times. | Visuospatial |
| The ability to anticipate weather changes and plan accordingly (i.e., bring a coat or umbrella) | Planning |
| Thinking ahead | Planning |
| Keeping living and work space organized. | Organizing |
| Balancing the checkbook without error | Organizing |
| The ability to do two things at once | Divided attention |
| Cooking or working and talking at the same time. | Divided attention |

**Appendix 2A**: Performance of the average ECog-12 at each cutoff point to detect cognitive impaired from cognitively unimpaired participants

| Cut-off point | Youden index | Sensitivity | Specificity | AUC | PPV | NPV | accuracy  (95%CI) |
| --- | --- | --- | --- | --- | --- | --- | --- |
| Self report |  |  |  |  |  |  |  |
| ≥ 1.16 | 0.21 | 0.92 | 0.29 | 0.61  (0.59-0.62) | 0.64 | 0.72 | 0.66  (0.63-0.68) |
| ≥ 1.26 | 0.35 | 0.81 | 0.54 | 0.68  (0.66-0.7) | 0.71 | 0.68 | 0.7  (0.67-0.72) |
| **≥ 1.36** | **0.41** | **0.75** | **0.66** | **0.7**  **(0.68-0.73)** | **0.75** | **0.65** | **0.71**  **(0.69-0.73)** |
| ≥ 1.46 | 0.38 | 0.65 | 0.73 | 0.69  (0.67-0.71) | 0.77 | 0.6 | 0.68  (0.66-0.71) |
| ≥ 1.56 | 0.38 | 0.59 | 0.79 | 0.69  (0.67-0.71) | 0.79 | 0.58 | 0.67  (0.65-0.7) |
| Study-partner report |  |  |  |  |  |  |  |
| ≥ 1.3 | 0.56 | 0.78 | 0.78 | 0.77  (0.75-0.79) | 0.83 | 0.71 | 0.78  (0.76-0.8) |
| ≥ 1.4 | 0.56 | 0.72 | 0.84 | 0.78  (0.76-0.8) | 0.87 | 0.68 | 0.77  (0.75-0.79) |
| **≥ 1.45** | **0.57** | **0.67** | **0.89** | **0.78**  **(0.76-0.8)** | **0.9** | **0.66** | **0.76**  **(0.74-0.79)** |
| ≥ 1.5 | 0.56 | 0.66 | 0.9 | 0.78  (0.76-0.8) | 0.9 | 0.65 | 0.76  (0.74-0.78) |
| ≥ 1.6 | 0,52 | 0.58 | 0.94 | 0.76  (0.74-0.78) | 0.93 | 0.61 | 0.73  (0.71-0.75) |

**Appendix 2B:** Performance of ECog-12 and memory concern question in detecting cognitive impaired vs cognitively unimpaired participants

|  | Youden index | Sensitivity | Specificity | AUC  (95%CI) | PPV | NPV | Accuracy  (95%CI) |
| --- | --- | --- | --- | --- | --- | --- | --- |
| ECog12 of pt  at CP ≥ 1.36 | 0.41 | 0.75 | 0.66 | 0.7  (0.68-0.73) | 0.75 | 0.65 | 0.71  (0.69-0.73) |
| ECog12 of pt at CP ≥ 1.36 + concern vs none of both | 0.59 | 0.93 | 0.66 | 0.79  (0.77-0.82) | 0.79 | 0.87 | 0.81  (0.79-0.84) |
| Any consistent SCD ECog12 of pt | 0.38 | 0.72 | 0.66 | 0.69  (0.67-0.71) | 0.75 | 0.63 | 0.69  (0.67-0.72) |
| Any occasional + consistent SCD ECog12 of pt | 0.11 | 0.97 | 0.14 | 0.55  (0.54-0.57) | 0.61 | 0.76 | 0.62  (0.6-0.65) |
| Any consistent SCD ECog-12 + memory concern vs none of both | 0.58 | 0.92 | 0.66 | 0.79  (0.77-0.82) | 0.78 | 0.87 | 0.81  (0.78-0.83) |
| Any consistent SCD ECog-12 + memory concern vs others* | 0.45 | 0.69 | 0.76 | 0.73  (0.7-0.75) | 0.78 | 0.67 | 0.73  (0.7-0.75) |
| ECog-12 sp  at CP ≥ 1.45 | 0.57 | 0.67 | 0.89 | 0.78  (0.76-0.8) | 0.9 | 0.66 | 0.76  (0.74-0.79) |
| Any consistent SCD ECog-12 sp | 0.55 | 0.7 | 0.85 | 0.78  (0.76-0.8) | 0.87 | 0.87 | 0.76  (0.74-0.78) |
| Any occasional + consistent SCD ECog-12 sp | 0.34 | 0.94 | 0.40 | 0.67  (0.65-0.69) | 0.69 | 0.83 | 0.72  (0.69-0.74) |

Note: CP = cutpoint, pt = participant, sp = study partner

*Others: Participants included those without any consistent SCD in ECog12 and memory concerns, as well as those with only one of each.

**Appendix 3A**: Performance of the average ECog-12 at each cutoff point to detect MCI from cognitively unimpaired participants

| Cut-off point | Youden index | Sensitivity | Specificity | AUC  (95%CI) | PPV | NPV | Accuracy  (95%CI) |
| --- | --- | --- | --- | --- | --- | --- | --- |
| Self-participants |  |  |  |  |  |  |  |
| ≥ 1.36 | 0.39 | 0.73 | 0.66 | 0.693 (0.67-0.72) | 0.69 | 0.69 | 0.69  (0.67-0.72) |
| **≥ 1.37** | **0.39** | **0.72** | **0.67** | **0.695 (0.67-0.72)** | **0.7** | **0.69** | **0.7**  **(0.67-0.72)** |
| ≥ 1.38 | 0.39 | 0.72 | 0.67 | 0.694 (0.67-0.72) | 0.7 | 0.69 | 0.69  (0.67-0.72) |
| ≥ 1.39 | 0.39 | 0.72 | 0.67 | 0.694 (0.67-0.72) | 0.7 | 0.67 | 0.69  (0.67-0.72) |
| Study-partner |  |  |  |  |  |  |  |
| ≥ 1.1 | 0.43 | 0.84 | 0.59 | 0.72  (0.69-0.74) | 0.69 | 0.78 | 0.72  (0.7-0.75) |
| ≥ 1.2 | 0.47 | 0.77 | 0.7 | 0.73  (0.71-0.75) | 0.73 | 0.74 | 0.73  (0.71-0.76) |
| **≥ 1.27** | **0.49** | **0.73** | **0.76** | **0.75**  **(0.72-0.76)** | **0.76** | **0.73** | **0.74**  **(0.73-0.77)** |
| ≥ 1.3 | 0.49 | 0.71 | 0.78 | 0.74  (0.72-0.76) | 0.77 | 0.71 | 0.74  (0.72-0.76) |
| ≥ 1.4 | 0.49 | 0.64 | 0.85 | 0.74  (0.72-0.76) | 0.82 | 0.69 | 0.74  (0.72-0.76) |

**Appendix 3B:** Performance of ECog-12 and memory concern question in detecting MCI vs cognitively unimpaired participants

|  | Youden index | Sensitivity | Specificity | AUC  (95%CI) | PPV | NPV | accuracy  (95%CI) |
| --- | --- | --- | --- | --- | --- | --- | --- |
| ECog12 of pt at CP ≥ 1.37 | 0.39 | 0.72 | 0.67 | 0.69  (0.68-0.72) | 0.7 | 0.69 | 0.695  (0.67-0.72) |
| ECog12 of pt at CP ≥ 1.37 + concern vs none of both | 0.59 | 0.93 | 0.66 | 0.795  (0.77-0.82) | 0.72 | 0.91 | 0.79  (0.76-0.82) |
| Any consistent SCD ECog12 of pt | 0.36 | 0.7 | 0.66 | 0.68  (0.66-0.71) | 0.69 | 0.68 | 0.68  (0.66-0.71) |
| Any occasional + consistent SCD ECog12 of pt | 0.11 | 0.97 | 0.14 | 0.55  (0.54-0.57) | 0.54 | 0.81 | 0.57  (0.54-0.59) |
| Any consistent SCD ECog-12 of pt + memory concern vs none of both | 0.59 | 0.93 | 0.66 | 0.8  (0.77-0.82) | 0.72 | 0.91 | 0.79  (0.76-0.82) |
| Any consistent SCD ECog-12 of pt + memory concern vs others* | 0.45 | 0.69 | 0.76 | 0.72  (0.67-0.75) | 0.72 | 0.74 | 0.73  (0.7-0.75) |
| ECog-12 sp at CP ≥ 1.27 | 0.49 | 0.73 | 0.76 | 0.75  (0.72-0.77) | 0.76 | 0.73 | 0.74  (0.72-0.76) |
| Any consistent SCD ECog-12 sp | 0.47 | 0.62 | 0.85 | 0.74  (0.71-0.76) | 0.82 | 0.68 | 0.73  (0.71-0.76) |
| Any occasional + consistent SCD ECog-12 sp | 0.32 | 0.92 | 0.40 | 0.66  (0.64-0.68) | 0.62 | 0.83 | 0.67  (0.64-0.7) |

Note: CP = cutpoint, pt = patient, sp = study partner

*Others: Participants included those without any consistent SCD in ECog12 and memory concerns, as well as those with positive only one of each.

**Appendix 4A**: Performance of the average ECog-12 at each cutoff point to detect dementia from MCI participants

| Cut-off point | Youden index | Sensitivity | Specificity | AUC  (95%CI) | PPV | NPV | accuracy  (95%CI) |
| --- | --- | --- | --- | --- | --- | --- | --- |
| Self-participants |  |  |  |  |  |  |  |
| ≥ 1.6 | 0.12 | 0.63 | 0.49 | 0.56  (0.52-0.6) | 0.28 | 0.81 | 0.52  (0.49-0.55) |
| **≥ 1.7** | **0.14** | **0.57** | **0.56** | **0.57**  **(0.53-0.61)** | **0.29** | **0.81** | **0.57**  **(0.53-0.6)** |
| ≥ 1.8 | 0.12 | 0.51 | 0.61 | 0.56  (0.52-0.6) | 0.29 | 0.8 | 0.58  (0.55-0.62) |
| Study-partners |  |  |  |  |  |  |  |
| ≥ 1.7 | 0.49 | 0.91 | 0.58 | 0.75  (0.72-0.77) | 0.41 | 0.96 | 0.66  (0.63-0.69) |
| ≥ 1.8 | 0.54 | 0.91 | 0.63 | 0.77  (0.74-0.79) | 0.45 | 0.95 | 0.72  (0.63-0.69) |
| **≥ 1.9** | **0.54** | **0.87** | **0.68** | **0.77**  **(0.74-0.8)** | **0.47** | **0.94** | **0.72**  **(0.69-0.75)** |
| ≥ 2 | 0.54 | 0.83 | 0.71 | 0.77  (0.74-0.8) | 0.47 | 0.93 | 0.74  (0.71-0.76) |

**Appendix 4B:** Performance of ECog-12 and memory concern question in detecting dementia vs MCI participants

|  | Youden index | Sensitivity | Specificity | AUC  (95%CI) | PPV | NPV | accuracy  (95%CI) |
| --- | --- | --- | --- | --- | --- | --- | --- |
| ECog12 of pt at CP ≥ 1.7 | 0.13 | 0.57 | 0.56 | 0.57  (0.53-0.61) | 0.29 | 0.81 | 0.57  (0.53-0.6) |
| ECog12 of pt at CP ≥ 1.7 + concern | -0.01 | 0.85 | 0.14 | 0.49  (0.46-0.53) | 0.33 | 0.64 | 0.37  (0.33-0.42) |
| Any consistent SCD ECog12 of pt | 0.06 | 0.76 | 0.3 | 0.53  (0.49-0.56) | 0.25 | 0.8 | 0.41  (0.37-0.44) |
| Any occasional + consistent SCD ECog12 of pt | 0 | 0.97 | 0.03 | 0.5  (0.49-0.51) | 0.24 | 0.76 | 0.25  (0.23-0.28) |
| Any consistent SCD ECog-12 of pt + memory concern vs none of both | -0.03 | 0.9 | 0.07 | 0.49  (0.46-0.51) | 0.29 | 0.64 | 0.32  (0.28-0.36) |
| Any consistent SCD ECog-12 + memory concern vs others* | 0.02 | 0.71 | 0.31 | 0.51  (0.48-0.55) | 0.29 | 0.74 | 0.43  (0.39-0.46) |
| ECog-12 sp at CP ≥ 1.9 | 0.53 | 0.86 | 0.67 | 0.77  (0.74-0.8) | 0.45 | 0.94 | 0.72  (0.69-0.75) |
| Any Consistent SCD ECog-12 sp | 0.33 | 0.95 | 0.38 | 0.66  (0.64-0.68) | 0.32 | 0.96 | 0.51  (0.48-0.55) |
| Any occasionally + consistently SCD ECog-12 sp | 0.07 | 0.99 | 0.08 | 0.54  (0.53-0.55) | 0.25 | 0.98 | 0.3  (0.27-0.33) |

Note: CP = cutpoint, pt = patient, sp = study partner

*Others: Participants included those without any consistent SCD in ECog12 and memory concerns, as well as those with only a positive one of each.

**Appendix 5A**: Performance of the average ECog-12 at each cutoff point to detect cognitive impairment (dementia and MCI) with amyloid pathology vs other participants (CU and CI without amyloid pathology)

| Cut-off point | Youden index | Sensitivity | Specificity | AUC  (95%CI) | PPV | NPV | accuracy  (95%CI) |
| --- | --- | --- | --- | --- | --- | --- | --- |
| Self-participants |  |  |  |  |  |  |  |
| ≥ 1.3 | 0.26 | 0.83 | 0.43 | 0.63  (0.61-0.65) | 0.44 | 0.82 | 0.57  (0.54-0.59) |
| **≥ 1.36** | **0.3** | **0.78** | **0.53** | **0.65**  **(0.63-0.68)** | **0.47** | **0.82** | **0.61**  **(0.59-0.64)** |
| ≥ 1.4 | 0.29 | 0.76 | 0.53 | 0.65  (0.62-0.67) | 046 | 0.83 | 0.61  (0.59-0.64) |
| Study-partners |  |  |  |  |  |  |  |
| ≥ 1.3 | 0.46 | 0.85 | 0.61 | 0.73  (0.71-0.75) | 0.54 | 0.88 | 0.7  (0.67-0.72) |
| ≥ 1.4 | 0.49 | 0.77 | 0.74 | 0.74  (0.72-0.76) | 0.57 | 0.87 | 0.72  (0.7-0.75) |
| **≥ 1.45** | **0.51** | **0.77** | **0.74** | **0.75**  **(0.73-0.78)** | **0.62** | **0.87** | **0.75**  **(0.72-0.77)** |
| ≥ 1.5 | 0.49 | 0.75 | 0.74 | 0.75  (0.73-0.77) | 0.61 | 0.85 | 0.75  (0.72-0.77) |

**Appendix 5B**: Performance of ECog-12 and memory concern question in detecting cognitive impairment (dementia and MCI) with amyloid pathology vs other participants (CU and CI without amyloid pathology)

|  | Youden index | Sensitivity | Specificity | AUC  (95%CI) | PPV | NPV | accuracy  (95%CI) |
| --- | --- | --- | --- | --- | --- | --- | --- |
| ECog12 of pt at CP ≥ 1.36 | 0.31 | 0.78 | 0.53 | 0.65  (0.63-0.68) | 0.47 | 0.82 | 0.61  (0.59-0.64) |
| ECog12 of pt at CP ≥ 1.36 + concern | 0.42 | 0.95 | 0.47 | 0.71  (0.69-0.73) | 0.51 | 0.94 | 0.65  (0.61-0.67) |
| Any consistent SCD ECog12 of pt | 0.3 | 0.75 | 0.55 | 0.65  (0.62-0.67) | 0.47 | 0.8 | 0.62  (0.59-0.64) |
| Any occasional + consistent SCD ECog12 of pt | 0.09 | 0.98 | 0.11 | 0.54  (0.53-0.56) | 0.37 | 0.91 | 0.41  (0.39-0.44) |
| Any consistent SCD ECog-12 + memory concern vs none of both | 0.42 | 0.93 | 0.49 | 0.71  (0.69-0.74) | 0.51 | 0.93 | 0.65  (0.62-0.68) |
| Any consistent impaired ECog-12 + memory concern vs others* | 0.36 | 0.72 | 0.64 | 0.68  (0.66-0.71) | 0.51 | 0.82 | 0.67  (0.64-0.69) |
| ECog-12 sp at CP ≥ 1.45 | 0.51 | 0.77 | 0.74 | 0.75  (0.73-0.78) | 0.61 | 0.87 | 0.75  (0.73-0.78) |
| Any consistent SCD ECog-12 sp | 0.47 | 0.78 | 0.69 | 0.74  (0.72-0.76) | 0.58 | 0.86 | 0.73  (0.7-0.75) |
| Any occasional + consistent SCD ECog-12 sp | 0.26 | 0.97 | 0.29 | 0.63  (0.61-0.65) | 0.42 | 0.94 | 0.53  (0.5-0.55) |

Note: CP = cutpoint, pt = patient, sp = study partner

*Others: Participants included those without any consistent SCD in ECog12 and memory concerns, as well as those with positive only one of each.

**Appendix 6A**: Performance of the average ECog-12 at each cutoff point to detect dementia from cognitively unimpaired participants

| Cut-off point | Youden index | Sensitivity | Specificity | AUC  (95%CI) | PPV | NPV | accuracy  (95%CI) |
| --- | --- | --- | --- | --- | --- | --- | --- |
| Self-participants |  |  |  |  |  |  |  |
| ≥ 1.3 | 0.85 | 0.84 | 0.55 | 0.69  (0.66-0.72) | 0.38 | 0.91 | 0.62  (0.58-0.65) |
| ≥ 1.4 | 0.87 | 0.79 | 0.67 | 0.73  (0.69-0.76) | 0.44 | 0.9 | 0.7  (0.66-0.73) |
| **≥ 1.43** | **0.86** | **0.76** | **0.73** | **0.74**  **(0.71-0.77)** | **0.48** | **0.9** | **0.73**  **(0.7-0.76)** |
| ≥ 1.5 | 0.87 | 0.74 | 0.73 | 0.73  (0.7-0.77) | 0.47 | 0.89 | 0.73  (0.7-0.76) |
| Study-partner |  |  |  |  |  |  |  |
| ≥ 1.5 | 0.85 | 0.95 | 0.9 | 0.92  (0.91-0.94) | 0.76 | 0.98 | 0.91  (0.89-0.93) |
| ≥ 1.6 | 0.87 | 0.93 | 0.94 | 0.93  (0.91-0.95) | 0.85 | 0.97 | 0.94  (0.92-0.95) |
| **≥ 1.67** | **0.87** | **0.93** | **0.95** | **0.93**  **(0.91-0.95)** | **0.87** | **0.97** | **0.95**  **(0.93-0.96)** |

**Appendix 6B:** Performance of ECog-12 and memory concern question in detecting dementia vs cognitively unimpaired participants

|  | Youden index | Sensitivity | Specificity | AUC  (95%CI) | PPV | NPV | accuracy  (96%CI) |
| --- | --- | --- | --- | --- | --- | --- | --- |
| ECog12 of pt at CP ≥ 1.43 | 0.49 | 0.76 | 0.73 | 0.74  (0.71-0.77) | 0.48 | 0.9 | 0.73  (0.7-0.76) |
| ECog12 of pt at CP ≥ 1.43 + concern | 0.61 | 0.9 | 0.71 | 0.81  (0.78-0.84) | 0.55 | 0.95 | 0.76  (0.73-0.8) |
| Any consistent SCD ECog12 of pt | 0.42 | 0.76 | 0.66 | 0.71  (0.68-0.74) | 0.43 | 0.89 | 0.69  (0.66-0.72) |
| Any occasional + consistent SCD ECog12 of pt | 0.11 | 0.97 | 0.14 | 0.55  (0.54-0.57) | 0.27 | 0.93 | 0.35  (0.31-0.38) |
| Any consistent SCD ECog-12 + memory concern vs none of both | 0.56 | 0.9 | 0.66 | 0.78  (0.75-0.82) | 0.51 | 0.95 | 0.73  (0.69-0.76) |
| Any consistent SCD ECog-12 + memory concern vs others* | 0.47 | 0.71 | 0.76 | 0.74  (0.7-0.77) | 0.51 | 0.89 | 0.75  (0.72-0.78) |
| ECog-12 sp at CP ≥ 1.67 | 0.87 | 0.93 | 0.95 | 0.93  (0.91-0.95) | 0.87 | 0.97 | 0.95  (0.93-0.96) |
| Any consistent SCD ECog-12 sp | 0.8 | 0.95 | 0.85 | 0.9  (0.88-0.92) | 0.68 | 0.98 | 0.87  (0.85-0.9) |
| Any occasional + consistent SCD ECog-12 sp | 0.39 | 0.99 | 0.40 | 0.7  (0.68-0.72) | 0.36 | 0.99 | 0.55  (0.52-0.58) |

Note: CP = cutpoint, pt = patient, sp = study partner

*Others: Participants included those without any consistent SCD in ECog12 and memory concerns, as well as those with only a positive one of each.

**Appendix 7: Performance of ECog-39 to differentiate cognitive statuses**

|  | **CI vs CU**  AUC (95%CI) | **MCI vs CU**  AUC (95%CI) | **Dementia vs CU**  AUC (95%CI) | **Dementia vs MCI**  AUC (95%CI) | **CI with amyloid pathology vs others (CU&CI without amyloid pathology)**  AUC (95%CI) |
| --- | --- | --- | --- | --- | --- |
| ECog-39 of pt* | CP ≥ 1.44  AUC = 0.71  (0.69-0.73) | CP ≥ 1.44  AUC = 0.71 (0.68-0.73) | CP ≥ 1.54  AUC =0.74  (0.70-0.77) | CP ≥ 1.62  AUC = 0.55 (0.52-0.59) | CP ≥ 1.32  AUC = 0.65 (0.63-0.67) |
| ECog-39 of pt with memory concern ** | AUC = 0.8 (0.78-0.83) | AUC = 0.8 (0.78-0.83) | AUC = 0.82 (0.79-0.86) | AUC = 0.49 (0.46-0.52) | AUC = 0.69 (0.67-0.72) |
| ECog-39 of sp* | CP ≥ 1.44  AUC = 0.79 (0.77-0.81) | CP ≥ 1.23  AUC = 0.76 (0.73-0.78) | CP ≥ 1.64  AUC = 0.95 (0.93-0.96) | CP ≥ 2.26  AUC = 0.79 (0.77-0.82) | CP ≥ 1.64  AUC = 0.76 (0.74-0.78) |

CP, cutpoint; AUC, Area Under the Curve of the “Receiver Operating Characteristic” curve; pt , self-participant; sp, study partner; CI, cognitive impaired participants; CU, cognitively unimpaired participants

***** Dichotomous score on each cutpoint

** Performance of ECog-12 combined with positive concern question compared with none of high ECog and memory concern
